# Association between smoking status and health-related quality of life: a study on differences among age groups

**DOI:** 10.3389/fpubh.2024.1508236

**Published:** 2025-01-08

**Authors:** Guanping Zhong, Yang Shu, Hongwei Li, Yuelin Zhou, Qiaoying Wei, Biao Yang, Lian Yang

**Affiliations:** ^1^HEOA Group, School of Public Health, Chengdu University of Traditional Chinese Medicine, Chengdu, China; ^2^HEOA Group, School of Management, Chengdu University of Traditional Chinese Medicine, Chengdu, China

**Keywords:** smoking status, EQ-5D-5L utility index, HRQOL, EQ-VAS, multiperson group

## Abstract

**Purpose:**

This study explored the effect of four different smoking statuses (non-smokers, moderate smokers, heavy smokers, and former smokers) on health-related quality of life (HRQOL) among residents aged 15 years and older in Sichuan Province, China with consideration of potential differences among age groups (young, middle-aged, and older adults).

**Methods:**

The EQ-5D-5L utility index and EQ-VAS score were used to measure HRQOL. Self-reporting and salivary cotinine test were used to determine the smoking status of respondents, and the Tobit regression model was used to explore the relationship between smoking status and HRQOL.

**Results:**

The Tobit regression model found a significant correlation between smoking status and HRQOL. Heavy smokers reported a lower EQ-VAS score compared to non-smokers (*p* < 0.01); the EQ-5D-5L utility index and EQ-VAS score reported by former smokers were lower compared to non-smokers (*p* < 0.05 and *p* < 0.01, respectively). In the young population, the EQ-VAS score of heavy smokers was lower than that of non-smokers (*p* < 0.05); In the middle-aged population, the EQ-VAS score of heavy smokers was lower than that of non-smokers (*p* < 0.05). The EQ-5D-5L utility index and EQ-VAS score of former smokers were lower than that of non-smokers (p < 0.05 and *p* < 0.001, respectively). However, in the older adult population, the EQ-5D-5L utility index and EQ-VAS score of moderate smokers were higher compared to non-smokers (*p* < 0.05 and *p* < 0.05, respectively).

**Conclusion:**

This study found a significant correlation between HRQOL and smoking status, with notable differences in the relationship between smoking, EQ-5D-5L utility index, and EQ-VAS scores across age groups. We recommend targeted measures to control tobacco use based on age, considering specific smoking risks for each group. In particular, attention should be paid to the harm of smoking among young and middle-aged groups, reduce the occurrence of smoking behavior through publicity and early intervention, and provide appropriate health interventions for the older adult group. In addition, effective smoking cessation support policies should be developed to encourage residents to quit or reduce smoking frequency, avoid the progression of moderate smoking to heavy smoking and thus lead to serious health threats.

## 1 Introduction

Smoking is one of the major health risk factors worldwide, widely reported for its harmful effects on individuals (1, 2). Smoking causes millions of deaths and a huge economic burden worldwide every year (3). Although the public has gradually recognized the dangers of smoking, it remains a serious public health challenge on a global scale.

Smoking is not only directly a hazard to health, but also directly affects the health-related quality of life (HRQOL) (4, 5). HRQOL is a multidimensional concept that includes physical health, mental health, and social functioning (6). This comprehensive self-assessment indicator of health status provides an evaluation of individuals’ physical condition, psychological function, social ability, and overall personal condition based on certain socioeconomic characteristics and cultural background and values. Indeed, previous studies have reported a significant correlation between quality of life and smoking status (7, 8). This is because smoking is associated with various non-communicable diseases and may have a negative impact on quality of life.

Developed countries’ studies show smoking significantly lowers HRQOL (9–11). Most of these studies have focused on populations from relatively homogeneous developed regions, and have not sufficiently accounted for the unique socio-economic and cultural contexts of developing countries. Although approximately 80% of the 1.3 billion tobacco users worldwide live in low-and middle-income countries, research in developing countries is limited. Therefore, studying the relationship between smoking and HRQOL in developing countries is crucial for improving smokers’ HRQOL. In China, although studies have explored the relationship between smoking and quality of life, the results are inconsistent. Some studies have found that smoking is associated with poorer HRQOL. For example, Luo et al. (12) observed that smokers had significantly lower EuroQol Visual Analogue Scale (EQ-VAS) score than non-smokers and Sun et al. (13) found that the EQ-VAS score of pregnant women reporting first-, second-, or third-hand smoke exposure during pregnancy were lower than those of pregnant women without tobacco exposure. Cheng et al. (14) further detailed that smoking was associated with a lower EQ-5D-5L utility index in Chinese people aged 40 years and older. In contrast, a study from Shandong Province, China indicated the relationship between smoking status and HRQOL varies across different age groups, with older adult smokers in their sample reporting a higher EQ-5D-3L utility index than non-smokers (15). This finding suggests that the impact of smoking on HRQOL may change with age, providing a new perspective for us to understand the relationship between smoking and HRQOL.

Although existing research in China has provided a preliminary understanding of the relationship between smoking and HRQOL, limitations remain. For example, these studies employed mostly single indicators to measure health utility and the scales used may have significant ceiling effects. In addition, smoking status information in the study only comes from self-reported data, which lacks objectivity and may introduce bias. Finally, the lack of information on the smoking years and intensity of current smokers may limit the stability of the results from these studies. Therefore, further research is warranted to comprehensively understand the impact of smoking on quality of life.

To mitigate the shortcomings of previous research, we employed the EQ-5D-5L scale, which is reported to have higher sensitivity and lower ceiling effects, along with the EQ-VAS score to comprehensively evaluate HRQOL. Further, we used self-report surveys and a salivary cotinine test to determine the smoking status of the survey subjects, obtain more objective smoking information. Moreover, smoking status was categorized based on smoking volume, allowing us to more comprehensively explore the relationship between smoking and HRQOL.

Sichuan Province, located in southwestern China, is a populous and economically developed region renowned for its high-quality tobacco cultivation. Under the influence of the region’s economic and cultural background, smoking is not merely a personal behavior but is deeply integrated into social interactions and daily life. According to the National Adult Tobacco Survey, the smoking rate among individuals aged 15 and above in Sichuan Province exceeded 25% in 2020. Against this backdrop, the research aimed to investigate smoking status and HRQOL in people aged 15 years and older in Sichuan Province, analyze the correlation between these two variables, and explore whether there were differences among different young, middle-aged, and older adult populations.

## 2 Methods

### 2.1 Data sources

A cross-sectional survey was conducted on a household basis in Sichuan Province, China from January 2022 to April 2023. We used a stratified multistage sampling method to extract samples. Firstly, after considering factors such as geographical location, population size, and economic level, four locations were selected as the survey areas: Wenjiang District of Chengdu City, Fushun County of Zigong City, Qingchuan County of Guangyuan City, and Xide County of Liangshan Prefecture. Subsequently, two streets and two townships were selected from each of the four regions, with two communities selected from each street and two villages selected from each township for investigation. A household survey was used to conduct face-to-face interviews with all residents who meet the inclusion criteria. To ensure the quality of the investigation, each investigator underwent strict training before the investigation. During the investigation process, a quality supervision team was established to implement quality control at every stage of the on-site investigation.

### 2.2 Sample

Research has shown that most individuals begin smoking after the age of 15 years (16), thus we only included individuals aged 15 years and older in our sample. The criteria for survey subjects included (1) being a resident in the sample area of Sichuan Province for at least 6 months and (2) voluntarily and independently agreeing to be interviewed. We excluded subjects who (1) were non-residents of Sichuan Province, (2) were aged <15 years, (3) were diagnosed with severe illnesses or intellectual disabilities, exhibited poor language expression, or declined to participate or cooperate with the investigation. During the survey period, 6,189 individuals aged 15 years and older participated in our study. To avoid estimation bias, questionnaires with missing values were considered invalid in the quality control program. This study included 5,723 samples, with an overall effective rate of 92.47%. We divided all survey subjects into three groups according to age, namely young population (15 ~ 44, *n* = 2,480), middle-aged adults (45 ~ 64, *n* = 1,883), and older adults (≥65, *n* = 1,360) (15, 17).

### 2.3 Research measurement

#### 2.3.1 Smoking status

We used both self-report surveys and salivary cotinine tests (18) to determine the smoking status of the survey subjects. Smoking Index (SI) was used to measure the smoking intensity of the current smokers—calculated as the number of cigarettes smoked per day * the number of years of smoking, where the number of years of smoking is the difference between current age and the age of regular smoking. Subjects with an SI value of <400 were defined as moderate smokers, whereas subjects with an SI value of ≥400 were defined as heavy smokers. Thus, a higher SI value indicates a more severe smoking habit. Notably, SI is only applicable to current smokers, who have smoked up to 100 cigarettes in their lifetime and have smoked within the 30 days prior to the survey. SI for current non-smokers was 0.

To verify the authenticity of self-reported smoking status, a saliva cotinine rapid detection kit (immunocolloidal gold with a sensitivity of 30 PPB) was used to measure the presence of cotinine in residents’ saliva on site. This test kit is a qualitative testing tool; a positive result indicates a current smoker, whereas a negative result indicates a current non-smoker. The on-site testing process was as follows: (1) subjects rinsed their mouths, (2) immediately following, the investigator used a collection cup to collect approximately 1 mL of the subject’s saliva, (3) a dropper was used to collect three drops of saliva from the cup (about 100 μL) and vertically drip it into the sample hole of the test paper, (4) test results were read after 5 min.

According to the above definition, non-smokers refer to those who report never attempting to smoke or smoking less than 100 cigarettes since birth and whose saliva is negative for cotinine. Former smokers are those who report smoking at least 100 cigarettes since birth, but have not smoked in the past 30 days, and have negative salivary cotinine levels. Moderate smokers refer to those who report smoking 100 or more cigarettes since birth, have smoked within the past 30 days, have positive salivary cotinine levels, and have an SI < 400. Heavy smokers are individuals who report smoking 100 or more cigarettes since birth, have smoked within the past 30 days, have positive salivary cotinine levels, and have an SI ≥ 400.

#### 2.3.2 Health-related quality of life

This study used the EQ-VAS and the EQ-5D-5L scales to measure HRQOL. The EQ-5D-5L includes five dimensions: mobility, self-care, usual activities, pain/discomfort, and anxiety/depression. Each dimension is divided into five levels: no problem, mild problem, moderate problem, serious problem, and extremely serious problem. EQ-VAS records individuals’ health status on a vertical scale. The scale ranges from 0 to 100, indicating the worst to best health state imagined by the respondents. In the survey, respondents were asked to mark a number on the scale to represent their current health level. Compared to the EQ-5D-3L scale, the EQ-5D-5L scale has improved measurement sensitivity, reduced ceiling effect, and can better reflect different health states (19). The Chinese version of EQ-5D-5L has been validated by domestic scholars and has good reliability and validity (20). The Cronbach’s *α* coefficient of this study was 0.795. To quantify HRQOL levels, the EQ-5D-5L utility index was calculated according to the EQ-5D score system for Chinese residents after converting the dimensional levels of the subjects’ answers (21). The range of the EQ-5D-5L utility index was −0.391 to 1, with higher scores indicating higher HRQOL levels.

#### 2.3.3 Covariate

Marital status was divided into three categories: single, married, divorced/widowed/other. Annual household income was divided into five equal groups, represented by Q1, Q2, Q3, Q4, and Q5, representing the low-income group, lower-middle-income group, middle-income group, upper-middle-income group, and high-income group, respectively. Education level was divided into three groups: elementary school and below, junior high school, and high school and above. Household registration is a registered residence system in Mainland China, which divides the population into urban population and rural population. Other covariates included age, gender, chronic disease, drink alcohol, medical visits within the past 2 weeks, and hospitalization within the past year. As studies have shown that retired residents have a lower quality of life (22), employment status was also included as a covariate in this study.

## 3 Statistical analysis

Statistical analysis was conducted using SPSS 24.0 and Stata 15.0. Frequency counts and composition ratios were used to describe the distribution of general demographic information and disease information of the survey subjects. Independent sample *t*-tests were used for mean comparison between the two groups, and one-way ANOVA was used for multi-group comparisons. When analyzing the influencing factors of HRQOL among the surveyed subjects, we used the Tobit regression model, with *p* < 0.05 indicating a statistically significant difference. Finally, to explore the age differences between smoking status and HRQOL, further stratified regression analysis was conducted by age group (young, middle-aged adults, and older adults).

## 4 Results

Of the 5,723 respondents, 4,231 were non-smokers (73.93%), 301 were former smokers (5.26%), 682 were moderate smokers (11.92%), and 509 were heavy smokers (8.89%). By population, young people (43.33%) were the main respondents, followed by middle-aged and older adults, accounting for 32.90 and 23.76%, respectively. The average EQ-5D-5L utility index of residents in the sample area was 0.946, and the average EQ-VAS score was 78.153, indicating the overall HRQOL was good.

Univariate analysis found the following variables had a statistically significant impact on the EQ-5D-5L utility index and EQ-VAS score of residents (*p* < 0.05): smoking status, age, gender, marital status, education level, annual household income, household registration type, whether they had a chronic disease, whether they had visited a doctor in the past 2 weeks, whether they had been hospitalized in the past year, whether they had drunk alcohol, and their employment status. In particular, the EQ-5D-5L utility index and EQ-VAS score of residents with rural household registration, hospitalization in the past year, medical visits in the past 2 weeks, and chronic diseases were lower than those with urban household registration, no hospitalization in the past year, no medical visits in the past 2 weeks, and no chronic diseases (*p* < 0.05). Notably, the EQ-5D-5L utility index and EQ-VAS score of residents who reported drinking alcohol were higher than those of non-drinkers. A more descriptive analysis of the basic information of the samples can be found in Table 1.

**Table 1:** EQ-5D-5L utility index, EQ-VAS score, and univariate analysis of residents with different characteristics.

Characteristic	*n* (%)	EQ-5D-5L Utility Index		EQ-VAS score	
		M ± Std	*P*	M ± Std	*P*
Total people	5,723	0.946 ± 0.109		78.153 ± 14.708	
Smoking status			<0.001		<0.001
Non-smokers	4,231(73.93)	0.946 ± 0.109		78.382 ± 15.068	
Moderate smokers	682(11.92)	0.968 ± 0.070		81.463 ± 12.166	
Heavy smokers	509(8.89)	0.946 ± 0.094		75.140 ± 13.220	
former smokers	301(5.26)	0.897 ± 0.171		72.518 ± 14.832	
Age (year)			<0.001		<0.001
15~	2,480(43.33)	0.984 ± 0.043		83.595 ± 12.371	
45~	1883(32.90)	0.945 ± 0.103		75.996 ± 14.188	
≥65	1,360(23.76)	0.878 ± 0.156		71.215 ± 15.629	
Gender			<0.001		<0.001
Female	3,296(57.59)	0.938 ± 0.115		77.110 ± 15.328	
Male	2,427(42.41)	0.956 ± 0.098		79.568 ± 13.699	
Marital status			<0.001		<0.001
Single	1,352(23.62)	0.983 ± 0.057		84.666 ± 13.000	
Married	3,957(69.14)	0.939 ± 0.113		76.444 ± 14.508	
Widowed/Divorced/Others	414(7.23)	0.887 ± 0.150		73.208 ± 15.394	
Education level			<0.001		<0.001
Elementary school and below	2073(36.22)	0.902 ± 0.141		72.922 ± 15.710	
Junior high school	1,493(26.09)	0.958 ± 0.096		78.978 ± 13.972	
High school and above	2,157(37.69)	0.980 ± 0.052		82.608 ± 12.459	
Annual household income			<0.001		<0.001
Q1	843(14.73)	0.907 ± 0.148		73.392 ± 16.324	
Q2	1,229(21.47)	0.934 ± 0.120		76.895 ± 15.188	
Q3	1,274(22.26)	0.947 ± 0.100		78.015 ± 14.333	
Q4	1,256(21.95)	0.963 ± 0.083		80.023 ± 13.616	
Q5	1,121(19.59)	0.967 ± 0.083		81.172 ± 13.374	
Household registration			<0.001		<0.001
Rural	3,675(64.21)	0.941 ± 0.114		77.481 ± 15.059	
Urban	2048(35.79)	0.954 ± 0.097		79.358 ± 13.979	
Chronic diseases			<0.001		<0.001
Yes	1762(30.79)	0.888 ± 0.148		70.023 ± 15.629	
No	3,961(69.21)	0.971 ± 0.072		81.769 ± 12.708	
Ever hospitalized			<0.001		<0.001
Yes	769(13.44)	0.889 ± 0.153		71.242 ± 15.923	
No	4,954(86.56)	0.955 ± 0.097		79.225 ± 14.214	
Ever medical visits			<0.001		<0.001
Yes	376(6.57)	0.875 ± 0.163		69.782 ± 17.887	
No	5,347(93.43)	0.951 ± 0.102		78.741 ± 14.277	
Drink alcohol			<0.001		<0.001
Yes	1,524(26.63)	0.961 ± 0.084		79.394 ± 13.020	
No	4,199(73.37)	0.940 ± 0.116		77.702 ± 15.251	
Employment status			<0.001		<0.001
Working	2,639(46.11)	0.959 ± 0.088		78.898 ± 13.455	
Retired	836(14.61)	0.918 ± 0.133		75.415 ± 14.230	
Unemployed	2,248(39.28)	0.940 ± 0.118		78.295 ± 16.114	

The one-way ANOVA results showed significant differences in the EQ-5D-5L utility index and EQ-VAS score among residents based on smoking status. Further comparison results from the Dunnett test (2-sided)^b^ showed that compared to non-smokers, moderate smokers had higher EQ-5D-5L utility index and EQ-VAS score, heavy smokers had lower EQ-VAS score, and former smokers had significantly lower EQ-5D-5L utility index and EQ-VAS score. After age stratification, it was found that the EQ-VAS score of heavy smokers in the young population was lower than that of non-smokers, the EQ-5D-5L utility index of moderate and heavy smokers in the older adult population were higher than that of non-smokers. No differences were found in the EQ-5D-5L utility index and EQ-VAS score among middle-aged people with different smoking statuses. More information can be found in Table 2.

**Table 2:** EQ-5D-5L utility index and EQ-VAS score of smoking status among different age groups.

Characteristic	*n*	EQ-5D-5L utility index	EQ-VAS score
		M ± Std	M ± Std
Total people	5,723	0.946 ± 0.109***^a^	78.153 ± 14.708***^b^
Smoking status			
Non-smokers	4,231(73.93)	0.946 ± 0.109	78.382 ± 15.068
Moderate smokers	682(11.92)	0.968 ± 0.070***^12^	81.463 ± 12.166***^12^
Heavy smokers	509(8.89)	0.946 ± 0.094	75.140 ± 13.220***^13^
Former smokers	301(5.26)	0.897 ± 0.171***^14^	72.518 ± 14.832***^14^
Young population (15~)	2,480	0.984 ± 0.043	83.595 ± 12.371*^b^
Smoking status			
Non-smokers	1952(78.71)	0.984 ± 0.044	83.643 ± 12.485
Moderate smokers	443(17.86)	0.983 ± 0.040	84.075 ± 11.026
Heavy smokers	58(2.34)	0.987 ± 0.031	79.310 ± 14.613*^13^
Former smokers	27(1.09)	0.992 ± 0.019	81.444 ± 17.656
Middle-aged population (45~)	1883	0.945 ± 0.103	75.996 ± 14.188
Smoking status			
Non-smokers	1,365(72.49)	0.942 ± 0.105	76.013 ± 14.610
Moderate smokers	146(7.75)	0.952 ± 0.092	77.726 ± 13.439
Heavy smokers	288(15.29)	0.957 ± 0.089	75.938 ± 12.834
Former smokers	84(4.46)	0.932 ± 0.122	72.905 ± 12.504
Older adult population (≥65)	1,360	0.878 ± 0.156***^a^	71.215 ± 15.629
Smoking status			
Non-smokers	914(67.21)	0.870 ± 0.158	70.685 ± 16.525
Moderate smokers	93(6.84)	0.921 ± 0.105**^12^	74.893 ± 11.325
Heavy smokers	163(11.99)	0.912 ± 0.108**^13^	72.245 ± 12.858
Former smokers	190(13.97)	0.868 ± 0.193	71.079 ± 14.970

*p*-values with statistical significance: *p < 0.05, **p < 0.01, ***p < 0.001.

^aOne-way ANOVA comparing EQ-5D-5L utility index based on smoking status.^

^bOne-way ANOVA comparing EQ-VAS score based on smoking status.^

^12^Dunnett test (2-sided)^b^ comparing non-smokers with moderate smokers.

^13^Dunnett test (2-sided)^b^ comparing non-smokers and heavy smokers.

^14^Dunnett test (2-sided)^b^ comparing non-smokers and former smokers.

In Table 3, after adjusting for age, gender, marital status, education level, annual household income, household registration, chronic disease, medical visits in the past 2 weeks, hospitalization in the past year, drink alcohol, and employment status, the Tobit regression model showed that smoking status was significantly related to EQ-5D-5L utility index and EQ-VAS score. The EQ-VAS score for heavy smokers was significantly lower compared with non-smokers (95% CI: −4.103 to −0.964, *p* < 0.01). The EQ-5D-5L utility index and EQ-VAS score of former smokers were also significantly lower compared to non-smokers (95% CI: −0.062 to −0.005, *p* < 0.05 and 95% CI: −5.030 to −1.299, *p* < 0.01, respectively).

**Table 3:** Tobit regression results of EQ-5D-5L utility index and EQ-VAS among residents based on smoking status.

Characteristic	EQ-5D-5L utility index				EQ-VAS score			
	Coefficient	SE	*P*	95%Cl	Coefficient	SE	*P*	95%Cl
Smoking status								
Non-smokers	Ref							
Moderate smokers	−0.019	0.012	0.114	(−0.042,0.005)	−0.187	0.694	0.787	(−1.548,1.173)
Heavy smokers	−0.003	0.013	0.837	(−0.028,0.023)	−2.533	0.801	0.002	(−4.103,−0.964)
former smokers	−0.033	0.015	0.022	(−0.062,−0.005)	−3.165	0.952	0.001	(−5.030,−1.299)
Age (year)								
15~	Ref							
45~	−0.057	0.010	<0.001	(−0.076,−0.037)	−3.383	0.583	<0.001	(−4.526,−2.239)
≥65	−0.139	0.012	<0.001	(−0.162,−0.116)	−6.441	0.743	<0.001	(−7.898,−4.984)
Gender								
Female	Ref							
Male	0.040	0.009	<0.001	(0.023,0.058)	2.670	0.512	<0.001	(1.666,3.675)
Marital status								
Single	Ref							
Married	−0.012	0.011	0.254	(−0.034,0.009)	−2.575	0.614	<0.001	(−3.779,−1.372)
Widowed/Divorced/Others	−0.039	0.015	0.010	(−0.068,−0.009)	−2.303	0.941	0.014	(−4.148,−0.459)
Education level								
Elementary school and below	Ref							
Junior high school	0.048	0.008	<0.001	(0.032,0.065)	1.909	0.529	<0.001	(0.873,2.946)
High school and above	0.052	0.010	<0.001	(0.032,0.072)	1.669	0.617	0.007	(0.460,2.878)
Annual household income								
Q1	Ref							
Q2	0.034	0.010	<0.001	(0.015,0.054)	2.364	0.638	<0.001	(1.114,3.614)
Q3	0.035	0.010	<0.001	(0.016,0.055)	1.964	0.647	0.002	(0.696,3.231)
Q4	0.031	0.011	0.003	(0.011,0.052)	1.737	0.671	0.010	(0.421,3.053)
Q5	0.025	0.011	0.028	(0.003,0.047)	1.746	0.700	0.013	(0.373,3.119)
Household registration								
Rural	Ref							
Urban	0.005	0.007	0.464	(−0.009,0.020)	0.832	0.439	0.058	(−0.029,1.693)
Chronic diseases								
Yes	Ref							
No	0.108	0.007	<0.001	(0.094,0.122)	8.060	0.464	<0.001	(7.151,8.969)
Ever hospitalized								
Yes	Ref							
No	0.057	0.008	<0.001	(0.041,0.074)	3.417	0.569	<0.001	(2.302,4.532)
Ever medical visits								
Yes	Ref							
No	0.106	0.011	<0.001	(0.084,0.128)	6.061	0.765	<0.001	(4.561,7.562)
Drink alcohol								
Yes	Ref							
No	−0.002	0.008	0.805	(−0.017,0.013)	0.142	0.464	0.760	(−0.768,1.052)
Employment status								
Working	Ref							
Retired	0.013	0.010	0.203	(−0.007,0.034)	2.968	0.676	<0.001	(1.643,4.293)
Unemployed	−0.014	0.007	0.058	(−0.028,0.000)	0.301	0.456	0.509	(−0.594,1.196)

Further, we explored the relationship between smoking status and EQ-5D-5L utility index and EQ-VAS score among different age groups. The results showed that among young people, the EQ-VAS score of heavy smokers was significantly lower than that of non-smokers (95% CI: −7.838 to −0.248, *p* < 0.05). In the middle-aged group, the EQ-VAS score of heavy smokers was significantly lower than that of non-smokers (95% CI: −5.361 to −0.606, *p* < 0.05). The EQ-5D-5L utility index and EQ-VAS score of former smokers were significantly lower than those of non-smokers (95% CI: −0.125 to-0.015, *p* < 0.05 and 95% CI: −9.473 to −2.747, *p* < 0.001, respectively). However, in the older adult population, the EQ-5D-5L utility index and EQ-VAS score of moderate smokers were higher compared to non-smokers (95% CI: 0.012 to 0.121, *p* < 0.05 and 95% CI: 0.692 to 8.074, *p* < 0.05, respectively). These results suggest that although smoking has an impact on the EQ-5D-5L utility index and EQ-VAS score of individuals in different age groups, the degree to which smoking affects health status varies with age. More information can be found in Table 4. More details are provided in Supplementary Table 1.

**Table 4:** Relationship between smoking status and EQ-5D-5L utility index and EQ-VAS score among different age groups.

Characteristic	EQ-5D-5L utility index				EQ-VAS score			
	Coefficient	SE	*P*	95%Cl	Coefficient	SE	*P*	95%Cl
Young population (15~)								
Smoking status								
Non-smokers	Ref							
Moderate smokers	−0.022	0.013	0.083	(−0.047, 0.003)	−0.124	0.868	0.886	(−1.826,1.578)
Heavy smokers	0.013	0.030	0.650	(−0.045, 0.072)	−4.043	1.935	0.037	(−7.838,−0.248)
Former smokers	0.040	0.045	0.371	(−0.048, 0.128)	−1.477	2.734	0.589	(−6.838,3.885)
Middle-aged population (45~)								
Smoking status								
Non-smokers	Ref							
Moderate smokers	−0.032	0.024	0.178	(−0.079, 0.015)	−0.841	1.418	0.553	(−3.622, 1.940)
Heavy smokers	−0.016	0.021	0.446	(−0.057, 0.025)	−2.984	1.212	0.014	(−5.361, −0.606)
Former smokers	−0.070	0.028	0.012	(−0.125, −0.015)	−6.110	1.715	<0.001	(−9.473, −2.747)
Older adult population (≥65)								
Smoking status								
Non-smokers	Ref							
Moderate smokers	0.066	0.028	0.016	(0.012, 0.121)	4.383	1.881	0.020	(0.692, 8.074)
Heavy smokers	0.002	0.023	0.936	(−0.044, 0.048)	−0.396	1.627	0.808	(−3.588, 2.796)
Former smokers	−0.009	0.022	0.687	(−0.051, 0.034)	−0.616	1.513	0.684	(−3.584, 2.353)

## 5 Discussion

To obtain more objective and authentic smoking information from participants, we employed both self-report and salivary cotinine testing to accurately evaluate the smoking behavior of residents. We explored the relationship between smoking and HRQOL among residents aged 15 and above in Sichuan Province and found that smoking had a significant impact on HRQOL. Compared to non-smokers, moderate smokers were likely to report higher EQ-5D-5L utility index and EQ-VAS score, while heavy smokers reported lower EQ-VAS score. The EQ-5D-5L utility index and EQ-VAS score of former smokers were also significantly lower than those of non-smokers. This discovery contradicts the findings of relevant studies in developed countries, which indicated mild and heavy smokers reported lower quality of life, and both mild and heavy smoking are significantly associated with higher mortality risks. There is a dose–response relationship in the negative association between smoking and HRQOL, with the results of heavy smokers being stronger than those of mild smokers (23–25). We speculate that this difference may be attributed to cultural differences, social backgrounds, and differences in tobacco cognition between different regions. In China, moderate smokers may believe that they do not smoke enough cigarettes to significantly impact their health, thus underestimating the harm of smoking to their health. In addition, in Chinese society, smoking is seen as a social tool and fashion (26, 27). Giving and receiving cigarettes are ways to gain social recognition and dignity and establish and maintain relationships with others. Moderate smokers may receive recognition and support from their social circle, thus holding a rational view of smoking and being more willing to believe that smoking will not negatively affect their health. In contrast, in some developed countries, smoking has been subject to strict control and social pressure. Most developed countries have implemented comprehensive tobacco control policies, including increasing taxes, media promotion, advertising bans, promoting smoke-free venues, and clear packaging warnings (28). Smoking has become a symbol of unhealthy behavior. In addition, the public has a high level of awareness of the harm of smoking to health, and people believe that smoking is harmful to health and regret starting smoking (29). Therefore, in these countries, mild smokers may also have a lower self-evaluation of their health status. These factors may lead to different attitudes toward smoking between China and developed countries, which partially explains the different results of smoking and HRQOL studies conducted in China from those of other countries.

In addition, the study also revealed that the association between smoking status and HRQOL varied among young, middle-aged, and older adults. Young, middle-aged heavy smokers have lower EQ-VAS scores vs. non-smokers. Previous studies have shown that heavy smokers are more susceptible to the negative effects of smoking, including a decrease in quality of life (25). The results of this study confirm this finding. We speculate that heavy smokers may be exposed to higher concentrations of nicotine and other tobacco components, and higher nicotine dependence levels are associated with a decrease in quality of life (30–32). Long-term heavy smoking causes various health issues. These health problems may lead to more physical and psychological discomfort and pain for heavy smokers as well as the need for more medical services and treatment, thereby increasing medical expenses and burdens. These factors may all reduce the life satisfaction and EQ-VAS score of heavy smokers. In this study, approximately 12% of smokers were non-heavy smokers. Considering that heavy smoking is more likely to bring about negative health effects, smoking cessation interventions should be targeted especially to new or moderate smokers to reduce dependence and addiction to tobacco, prevent new or moderate smokers from developing into heavy smokers, and reduce the incidence of smoking-related diseases and deaths.

In the middle-aged population, former smokers exhibited lower EQ-5D-5L utility index and EQ-VAS score. Firstly, former smokers may have already been suffering from serious health effects of smoking, including respiratory diseases, cardiovascular disease, and cancer, which have significantly reduced their quality of life. In addition, 40.6% of individuals who quit smoking did so because of illness, and 26.9% did so to prevent disease (33). As a result, previous smokers are more sensitive to their health status and may pay more attention to physical discomfort and health issues, which may result in lower scores in their quality-of-life evaluations. In addition, smoking-associated diseases have placed a huge burden on the economy worldwide, and this is also true in China. Treating smoking-related diseases requires a significant number of medical resources and costs, which puts significant financial pressure on individuals and their families and may further reduce their quality of life. The combined impact of these health and economic factors may lead to former smokers exhibiting lower levels of quality of life and overall health status.

In the older adult population, moderate smokers reported higher HRQOL than non-smokers. Previous studies have shown that older smokers often do not believe in the negative effects of smoking on their health. Older adults believe that they will not face health risks in the future due to smoking and daily cigarette consumption is safe (34). In other words, the older adult population is more inclined to hold various self-exemption beliefs (35). We speculate that this may be because those smokers with poorer physical fitness or higher smoking intensity may have died early in young or middle age (36), thus current surviving older moderate smokers tend to represent groups that have relatively good physical fitness and are more tolerant of smoking effects. This “survival bias” may allow moderate smokers to show better HRQOL observations in the HRQOL assessment. Meanwhile, for heavy smokers, serious health problems due to tobacco addiction may significantly reduce HRQOL, which further highlights the relative advantage of moderate smokers. Secondly, the social role of smoking under cultural norms may also be another important factor. In Chinese culture, smoking is not only an individual behavior, but also an important social tool, so smoking may have become a social habit in the older adult. Studies show that higher social participation is associated with higher smoking risk in older adults (37). Older adults who experience retirement may have less work or family responsibilities, allowing more time for social activities, and smoking may become a link in social activities, helping them alleviate social isolation and thereby improving mental health and overall HRQOL. Further studies showed that high social participation was associated with self-reported good health status and objective health status (38). Therefore, for the moderate older adult smokers, smoking may serve as a tool to maintain social relationships, indirectly promoting the mental health and HRQOL of the older adult.

Public health needs and lifestyle differences should be considered when developing public health policies for different age groups. For young and middle-aged groups, focus should be on early intervention to prevent smoking behaviors, intensive education on the dangers of smoking, and providing targeted cessation support services to reduce the negative health effects of early smoking. For older people, in addition to smoking cessation interventions, it is equally crucial to promote active participation in social activities, reduce social isolation, and enhance mental health and social support networks. By comprehensively considering age characteristics and health needs, formulating personalized public health interventions will effectively improve the quality of life of people of all ages and improve their overall health status.

## 6 Limitations

Our research has limitations. First, it’s based on a household survey in Sichuan, China, limiting generalization. Second, the cross-sectional design cannot establish a causal link between smoking and HRQOL. Third, “survival bias” should be considered as a limitation of the research. Smokers in poorer health are more likely to die prematurely, leaving behind relatively healthier individuals who are more resilient to smoking’s effects. This “survival bias” may result in older adult smokers displaying higher HRQOL scores in the study. In other words, the observed results may not fully reflect the long-term negative health effects of smoking. To address this limitation, future research could consider adopting a longitudinal study design to track the health changes of individuals with different smoking statuses, thereby better controlling for the impact of survival bias. Fourth, although the present study used self-report combined with the salivary cotinine test to assess smoking behavior, both approaches may still have a bias. Self-report may be influenced by recall bias or social expectation effects, and respondents may underestimate or overestimate their smoking behavior. Although the salivary cotinine test is a more objective assessment method, it mainly reflects recent smoking behavior and may not accurately capture long-term smoking habits, especially for occasional smokers. Lastly, we did not consider smoking abstinence duration. These should be addressed in future research to better understand the smoking-HRQOL relationship.

## 7 Conclusion

Our study found a significant association between smoking and HRQOL among Chinese residents aged 15+. This relationship varies by age. We recommend the governments take multi-level measures to control smoking, considering age-specific risks. Special attention should be paid to the harm of smoking in young and middle-aged groups, and the reduction of smoking behavior through publicity and early intervention, providing psychological and medical support for smokers. Provide appropriate health intervention for the older adult. Through more social activities, reduce the sense of social isolation, encourage older adult people to quit smoking as early as possible, and avoid moderate to heavy smoking. In addition, effective smoking cessation support policies should be developed to encourage residents to quit or reduce smoking frequency, this is the key to improving the public health and the HRQOL.

## Data Availability

The data analyzed in this study is subject to the following licenses/restrictions: the data underlying this article cannot be shared publicly due to the privacy of individuals that participated in the study. The data will be shared on reasonable request to the corresponding author. Requests to access these datasets should be directed to Lian Yang, yyanlian@163.com.
