# Supplementary material for: Association between smoking status and health-related quality of life: a study on differences among age groups
Source: Front Public Health. 2025 Jan 8;12:1508236. doi: 10.3389/fpubh.2024.1508236 (PMC11751219; doi:10.3389/fpubh.2024.1508236)
Supplement: Supplementary file 1 [file Data_Sheet_1.pdf]

Supplementary Fig 1. Frequency Distribution of EQ-5D-5L utility value

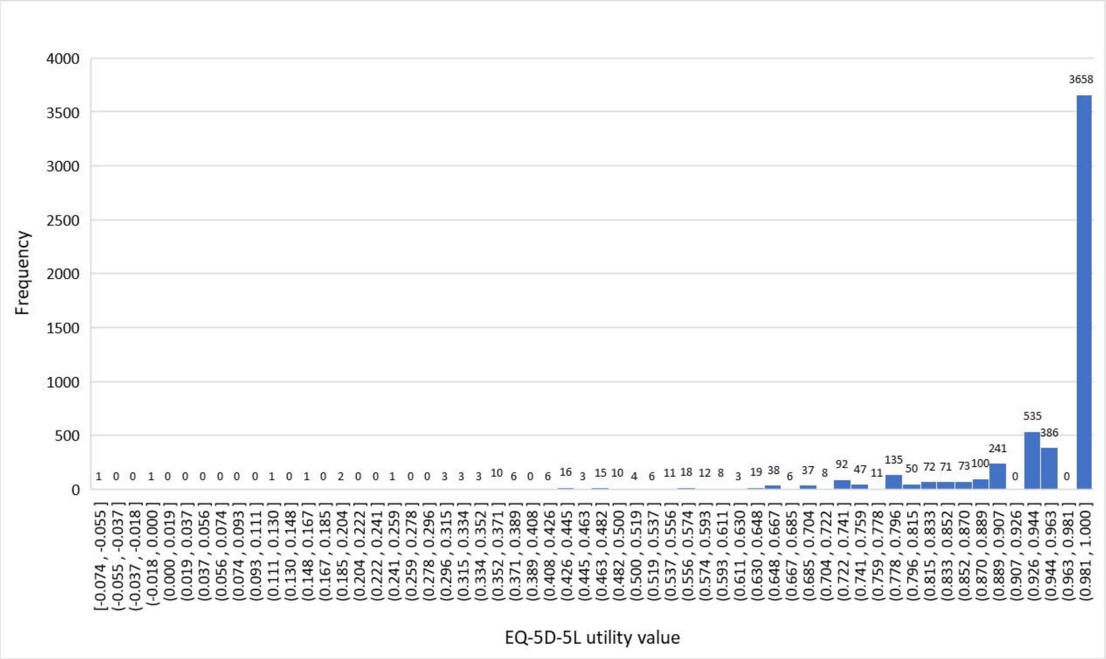

Supplementary Fig 2 .Frequency Distribution of EQ-VAS Scores

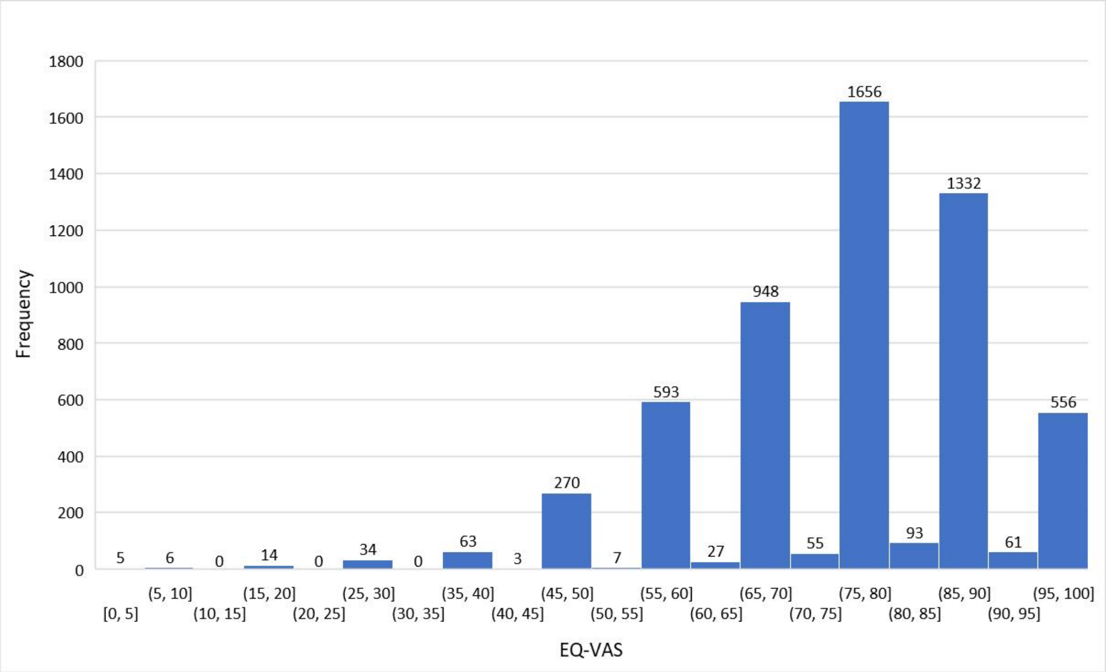

Supplementary Table 1 Tobit regression results on EQ-5D-5L utility index and EQ-VAS Score in the young population

Characteristic	EQ-5D-5L Utility Index				EQ-VAS score			
	Coefficient	SE	P	95%Cl	Coefficient	SE	P	95%Cl
Smoking status								
Nonsmokers					Ref			
Moderate smokers	-0.022	0.013	0.083	(-0.047,0.003)	-0.124	0.868	0.886	(-1.826,1.578)
Heavy smokers	0.013	0.030	0.650	(-0.045,0.072)	-4.043	1.935	0.037	(-7.838, -0.248)
former smokers	0.040	0.045	0.371	(-0.048,0.128)	-1.477	2.734	0.589	(-6.838,3.885)
Gender								
Female					Ref			
Male	0.032	0.010	0.002	(0.012,0.052)	3.025	0.669	< 0.001	(1.712,4.338)
Marital status								
Single					Ref			
Married	-0.009	0.010	0.369	(-0.029, 0.011)	-2.099	0.709	0.003	(-3.490,-0.708)
Widowed/Divorced/Others	-0.034	0.026	0.197	(-0.086,0.018)	-2.631	1.943	0.176	(-6.442,1.179)
Education level								
Elementary school and below					Ref			
Junior high school	0.030	0.016	0.053	(-0.0004,0.061)	1.548	1.083	0.153	(-0.577,3.672)
High school and above	0.025	0.015	0.101	(-0.005,0.054)	0.694	1.048	0.508	(-1.362,2.750)
Annual household income								
Q1					Ref			
Q2	0.013	0.019	0.492	(-0.024,0.049)	3.265	1.231	0.008	(0.851,5.678)
Q3	-0.008	0.017	0.643	(-0.042,0.026)	1.367	1.169	0.242	(-0.926,3.660)
Q4	-0.0001	0.017	0.993	(-0.034,0.034)	0.382	1.149	0.740	(-1.872,2.635)
Q5	-0.005	0.017	0.749	(-0.039,0.028)	0.999	1.146	0.383	(-1.247,3.246)
Household registration								
Rural					Ref			
Urban	-0.028	0.009	0.002	(-0.045,-0.010)	0.410	0.612	0.504	(-0.791,1.610)
Chronic diseases								
Yes					Ref			
No	0.083	0.013	< 0.001	(0.057,0.108)	8.432	1.007	< 0.001	(6.457,10.407)
Ever hospitalized								
Yes					Ref			
No	0.032	0.015	0.030	(0.003,0.061)	1.103	1.096	0.314	(-1.047,3.253)
Ever medical visits								
Yes					Ref			
No	0.062	0.016	< 0.001	(0.031,0.094)	4.087	1.264	0.001	(1.608,6.565)

Drink alcohol								
Yes					Ref			
No	0.022	0.009	0.020	(0.004,0.041)	1.194	0.654	0.068	(-0.090,2.477)
Employment status								
Working					Ref			
Retired	-0.047	0.066	0.481	(-0.176,0.083)	-0.165	5.195	0.975	(-10.352,10.023)
Unemployed	0.004	0.010	0.664	(-0.015,0.024)	2.264	0.675	0.001	(0.940,3.587)

Supplementary Table 2 Tobit regression results on EQ-5D-5L utility index and EQ-VAS score in middle-aged population

	EQ-5D-5L Utility Index				EQ-VAS score			
Characteristic	Coefficient	SE	P	95%Cl	Coefficient	SE	P	95%Cl
Smoking status								
Nonsmokers					Ref			
Moderate smokers	-0.032	0.024	0.178	(-0.079,0.015)	-0.841	1.418	0.553	(-3.622,1.940)
Heavy smokers	-0.016	0.021	0.446	(-0.057,0.025)	-2.984	1.212	0.014	(-5.361, -0.606)
former smokers	-0.070	0.028	0.012	(-0.125,-0.015)	-6.110	1.715	< 0.001	(-9.473,-2.747)
Gender								
Female					Ref			
Male	0.050	0.018	0.004	(0.016,0.085)	2.642	1.025	0.010	(0.632,4.652)
Marital status								
Single					Ref			
Married	-0.049	0.039	0.209	(-0.124, 0.027)	0.947	2.171	0.663	(-3.311,5.205)
Widowed/Divorced/Others	-0.082	0.044	0.059	(-0.168,0.003)	1.703	2.543	0.503	(-3.284,6.690)
Education level								
Elementary school and below					Ref			
Junior high school	0.039	0.012	0.001	(0.015,0.062)	0.568	0.739	0.442	(-0.881,2.017)
High school and above	0.053	0.017	0.003	(0.019,0.087)	1.829	1.035	0.077	(-0.200,3.858)
Annual household income								
Q1					Ref			
Q2	0.024	0.015	0.108	(-0.005,0.054)	0.370	0.956	0.699	(-1.505,2.245)
Q3	0.046	0.016	0.005	(0.014,0.077)	0.877	1.014	0.388	(-1.113,2.866)
Q4	0.038	0.017	0.025	(0.005,0.070)	2.077	1.044	0.047	(0.028,4.125)
Q5	0.037	0.020	0.056	(-0.001,0.076)	2.072	1.204	0.086	(-0.290,4.433)
Household								

registration								
Rural					Ref			
Urban	0.022	0.013	0.088	(-0.003,0.048)	0.386	0.786	0.623	(-1.156,1.929)
Chronic diseases								
Yes					Ref			
No	0.100	0.011	< 0.001	(0.079,0.121)	8.390	0.659	< 0.001	(7.097,9.682)
Ever hospitalized								
Yes					Ref			
No	0.043	0.014	0.002	(0.015,0.070)	4.685	0.901	< 0.001	(2.918,6.452)
Ever medical visits								
Yes					Ref			
No	0.128	0.018	< 0.001	(0.092,0.164)	7.881	1.266	< 0.001	(5.397,10.365)
Drink alcohol								
Yes					Ref			
No	-0.014	0.014	0.297	(-0.041,0.013)	-1.107	0.822	0.178	(-2.719,0.504)
Employment status								
Working					Ref			
Retired	-0.020	0.016	0.206	(-0.052,0.011)	1.032	0.980	0.292	(-0.890,2.955)
Unemployed	-0.027	0.012	0.023	(-0.050,-0.004)	-1.430	0.749	0.056	(-2.898,0.039)

Supplementary Table 3 Tobit regression results on EQ-5D-5L utility index and EQ-VAS score in older adults.

	EQ-5D-5L Utility Index				EQ-VAS score			
Characteristic	Coefficient	SE	P	95%Cl	Coefficient	SE	P	95%Cl
Smoking status								
Nonsmokers					Ref			
Moderate smokers	0.066	0.028	0.016	(0.012,0.121)	4.383	1.881	0.020	(0.692,8.074)
Heavy smokers	0.002	0.023	0.936	(-0.044,0.048)	-0.396	1.627	0.808	(-3.588, 2.796)
former smokers	-0.009	0.022	0.687	(-0.051,0.034)	-0.616	1.513	0.684	(-3.584,2.353)
Gender								
Female					Ref			
Male	0.010	0.017	0.549	(-0.024,0.045)	0.138	1.223	0.910	(-2.261,2.537)
Marital status								
Single					Ref			
Married	0.027	0.044	0.541	(-0.060, 0.114)	-1.980	3.210	0.537	(-8.278,4.317)
Widowed/Divorced/Others	0.010	0.046	0.823	(-0.080,0.101)	-1.131	3.328	0.734	(-7.660,5.397)

Education level								
Elementary school and below				Ref				
Junior high school	0.045	0.016	0.004	(0.014,0.076)	2.694	1.099	0.014	(0.537,4.851)
High school and above	0.103	0.023	< 0.001	(0.058,0.148)	4.208	1.525	0.006	(1.216,7.199)
Annual household income								
Q1				Ref				
Q2	0.052	0.017	0.002	(0.019,0.085)	4.085	1.195	0.001	(1.741,6.429)
Q3	0.045	0.017	0.010	(0.011,0.079)	3.810	1.250	0.002	(1.357,6.262)
Q4	0.036	0.021	0.086	(-0.005,0.078)	3.860	1.495	0.010	(0.926,6.793)
Q5	0.060	0.023	0.008	(0.015,0.105)	3.488	1.581	0.028	(0.386,6.591)
Household registration								
Rural				Ref				
Urban	0.048	0.014	0.001	(0.020,0.077)	2.886	1.014	0.005	(0.896,4.876)
Chronic diseases								
Yes				Ref				
No	0.119	0.013	< 0.001	(0.094,0.143)	6.750	0.863	< 0.001	(5.058,8.442)
Ever hospitalized								
Yes				Ref				
No	0.080	0.014	< 0.001	(0.054,0.107)	3.694	0.993	< 0.001	(1.746,5.642)
Ever medical visits								
Yes				Ref				
No	0.107	0.020	< 0.001	(0.069,0.146)	5.779	1.458	< 0.001	(2.919,8.639)
Drink alcohol								
Yes				Ref				
No	-0.044	0.015	0.004	(-0.074,-0.014)	-1.242	1.055	0.239	(-3.313,0.828)
Employment status								
Working				Ref				
Retired	-0.015	0.019	0.413	(-0.052,0.021)	0.742	1.290	0.566	(-1.789,3.272)
Unemployed	-0.037	0.016	0.022	(-0.069,-0.005)	-2.045	1.143	0.074	(-4.287,0.196)
